# Immune Gene Signatures and Immunotypes in Immune Microenvironment Are Associated With Glioma Prognose

**DOI:** 10.3389/fimmu.2022.823910

**Published:** 2022-04-14

**Authors:** Xiang-Xu Wang, Haiyan Cao, Yulong Zhai, Shi-Zhou Deng, Min Chao, Yaqin Hu, Yueyang Mou, Shaochun Guo, Wenjian Zhao, Chen Li, Yang Jiao, Guolian Xue, Liying Han, Hong-Mei Zhang, Liang Wang

**Affiliations:** ^1^ Department of Clinical Oncology, Xijing Hospital, Fourth Military Medical University, Xi’an, China; ^2^ Department of Neurosurgery, Tangdu Hospital, Fourth Military Medical University, Xi’an, China; ^3^ College of Life Sciences, Northwest University, Xi’an, China

**Keywords:** glioma, immunotype, immune microenvironment, macrophages, immunotherapy

## Abstract

Glioma is the most common primary malignant brain tumor in adults with very poor prognosis. The limited new therapeutic strategies for glioma patients can be partially attributed to the complex tumor microenvironment. However, knowledge about the glioma immune microenvironment and the associated regulatory mechanisms is still lacking. In this study, we found that, different immune subtypes have a significant impact on patient survival. Glioma patients with a high immune response subtype had a shorter survival compared with patients with a low immune response subtype. Moreover, the number of B cell, T cell, NK cell, and in particular, the macrophage in the immune microenvironment of patients with a high immune response subtype were significantly enhanced. In addition, 132 genes were found to be related to glioma immunity. The functional analysis and verification of seven core genes showed that their expression levels were significantly correlated with the prognosis of glioma patients, and the results were consistent at tissue levels. These findings indicated that the glioma immune microenvironment was significantly correlated with the prognosis of glioma patients and multiple genes were involved in regulating the progression of glioma. The identified genes could be used to stratify glioma patients based on immune subgroup analysis, which may guide their clinical treatment regimen.

## Introduction

Glioma is the most common malignant tumor of the central nervous system, belonging to the neuroepithelial group of tumors (1). According to the WHO classification guidelines for central nervous system tumors (2021 edition), gliomas are classified by IDH1/2 mutation and 1p/19q codeletion (2). At present, the treatment modes for glioma mainly include craniotomy and comprehensive therapeutics based on radiotherapy and chemotherapy. Even with such aggressive therapeutic strategy, the prognosis of gliomas remains unsatisfactory (3, 4). The dismal outcome may be attributed to the complex heterogeneity of gliomas, their invasive growth, and the difficulties associated with complete resection for protecting important neurological functions. In addition, The presence of the blood-brain barrier, making it difficult for macromolecular drugs to pass through (5, 6). At present, immunotherapy has shown significant therapeutic advantages in treatment for several cancer types. However, recent results of the phase 3 clinical trial CheckMate-143 were disappointing. There was no significant difference in median overall survival between nivolumab *vs.* bevacizumab administration in patients with recurrent GBM (7–9).

It has been reported that the infiltration abundance and functional state of immune cells in the glioma immune microenvironment are special, which may be significantly related to the distinct prognosis of glioma patients (10). Janet V Cross etc. (11) showed that a highly inhibitory tumor immune microenvironment can promote tumor growth. However, a systematic and comprehensive classification for different immune types to guide the evaluation of prognosis and treatment response remains lacking. The immune microenvironment of glioma contains several different types of immune cells, including macrophages, microglial, myeloid suppressors, dendritic, B cells, T cells, and NK cells (12, 13). A subtype of tumor-associated macrophages in the tumor microenvironment can promote tumor metastasis and resistance to chemotherapy (14). The interaction between various immune cells and their effects on tumor cells constitute a complex functional system. The complexity of the glioma immune microenvironment is induced by both the microenvironment of the central nervous system and by tumor cells. Elucidating the immune characteristics of the glioma immune microenvironment would help design targeted strategies. In addition, studying the composition of the glioma immune microenvironment and identifying specific immune-related genes and immune cell types in the microenvironment associated with glioma prognosis would contribute to our understanding of the molecular mechanisms underlying glioma immunosuppression (15).

Here, by exploring transcriptome data in The Chinese Glioma Genome Atlas (CGGA) (http://cgga.org.cn) and The Cancer Genome Atlas (TCGA) (http://cancergenome.nih.gov) database, we discovered that the glioma subtype with a high immune response had a shorter survival time compared with patients with a low immune response subtype. We further analyzed the genes associated with immune typing, seven genes related to patient prognosis and their corresponding characteristics were investigated accordingly. We constructed a prognostic prediction model for glioma immunotherapy and the findings may provide valuable information for developing targeted immunotherapy for glioma patients. The differences in tumor immune microenvironment and related differential gene expression may provide new options for designing individualized treatment regimens and identifying new targets for immunotherapy for glioma patients.

## Materials and Methods

### Glioma Datasets

Gene expression data and clinical information were retrospectively obtained from publicly available datasets in the CGGA (http://www.cgga.org.cn/) and TCGA databases (https://cancergenome.nih.gov/). A total of 1438 samples were enrolled in this analysis, including both the CGGA cohort (n=749) (**Table 1**) and TCGA cohort (n=689).

**Table 1:** Differences in clinical features among immune subtypes of glioma.

Variables	Overall	High	Low	p
	n = 749	n = 374	n = 375	
**Age [mean (SD)]**	43.26 (12.23)	45.71 (13.84	40.81 (9.81)	<0.001
**Risk_score [mean (SD)]**	1.47 (1.30)	2.44 (1.20)	0.49 (0.19)	<0.001
**OS_time [mean (SD)]**	3.22 (2.88)	1.80 (1.88)	4.64 (3.00)	<0.001
**Gender (%)**				0.678
Male	442 (59.0)	224 (59.9)	218 (58.1)	
Female	307 (41.0)	150 (40.1)	157 (41.9)	
**Grade (%)**				<0.001
Grade II	218 (29.1)	38 (10.2)	180 (48.0)	
Grade III	240 (32.0)	90 (24.1)	150 (40.0)	
Grade IV	291 (38.9)	246 (65.8)	45 (12.0)	
**IDH (%)**				<0.001
Wildtype	339 (45.3)	282 (75.4)	57 (15.2)	
Mutation	410 (54.7)	92 (24.6)	318 (84.8)	
**PRS_type (%)**				<0.001
Primary	502 (67.0)	220 (58.8)	282 (75.2)	
Recurrent	222 (29.6)	134 (35.8)	88 (23.5)	
Secondary	25 (3.3)	20 (5.3)	5 (1.3)	
**OS_status(%)**				<0.001
Dead	456 (60.9)	314 (84.0)	142 (37.9)	
Alive	293 (39.1)	60 (16.0)	233 (62.1)	

### Immune-Subgroup Identification and Verification

To estimate the immune status of each sample, a total of 25 immune-related gene sets were manually selected from previously published literature (16). We performed unsupervised clustering of glioma samples based on the ssGSEA scores for the 25 immune-related gene sets and identified three distinct immune subgroups. The ssGSEA score was calculated using the “GSVA” package in R. The “ConsensuClusterPlus” package was used to determine the number of stable clusters (17). The ESTIMATE algorithm [doi: 10.1038/ncomms3612] was used to analyze the Immune Score, Stromal Score, ESTIMATE Score, and tumor purity.

### Gene Set Variation Analysis (GSVA) for KEGG Enrichment and Hallmark Pathways

Gene set variation analysis (GSVA) algorithm was used to investigate the variations in pathways among the different immune-subgroups using the ‘GSVA’ package (18). The “c2.cp.kegg.v6.2.symbols” and “h.all.v7.2.symbols” gene sets from Molecular Signatures Database (MSigDB) (http://www.gsea-msigdb.org) were downloaded and used for the GSVA analysis.

### Comparisons of Fractions of Immune Cell Infiltrations

The deconvolution approach based on CIBERSORT (19) was used to estimate the abundances of 22 immune cell types based on their gene expression profiles and significant samples with an empirical CIBERSORT *P*<0.05 were selected for further comparison. We compared the fractions of 22 immune cell types among the three immune-subgroups using the Mann–Whitney U test.

### Selection of Immune-Subgroup Related Genes (IRGs)

CGGA data were correspondingly divided into the immune-H, immune-M, and immune-L clusters. According to the set values of *P*<0.05 and | log2FC |>1, we used the “limma” package to identify the differentially expressed genes (DEGs). The Venn diagram was drawn to identify overlapping IRGs from the above analyses. The DEGs among the IRGs in all three immune-subgroups were selected using the Venn diagram.

### Construction of a Prognostic Model Based on the IRGs

We used lasso and multivariate analyses to select the significant prognostic IRGs. We calculated the regression coefficients and hazard ratios (HRs) for each gene. Finally, the relevant mRNAs were identified. The prognostic risk score model for glioma patients was a weighted sum of each optimal prognosis mRNA expression and the relative regression coefficient calculated using the multivariate model. The risk score formula was as follows:


RiskScore= ∑i Coef (mRNAi) ∗ Exp(mRNAi)

All patients were divided into high- and low-risk groups based on the median risk score. The Kaplan–Meier survival curves of the two groups were plotted. ROC curves were plotted to evaluate the specificity and sensitivity of the model.

### Western Blotting (WB)

We selected eight pairs of glioma tissues including the carcinoma and peritumoral tissues for WB. Tissues were lysed with the RIPA buffer (Pierce, Rockford). The protein concentration was measured using a BCA protein assay kit (Pierce, Rockford). Equal amounts of total protein were loaded on SDS-PAGE gel, transferred onto poly vinylidene fluoride (PVDF) membranes (Millipore, Bedford) and then immunoblotted with the primary antibodies (Listed in **Table 2**) for 24h at 4°C. After washing with TBST buffer, PVDF membranes were incubated with secondary horseradish peroxidase-conjugated goat anti-mouse or anti-rabbit antibody (TDY BIOTEC, Beijing, China) and detected using an enhanced chemiluminescence system (Thermo Scientific, Rockford, IL), according to the manufacturer’s instructions. The expression of Actin was used as internal control.

**Table 2:** Primary antibodies used for western blot and immunohistochemistry.

Antibody	Company (Cat. No.)	Working Dilutions
SVOP	HUAAN (ER65055)	WB: 1/1000 IHC: 1/400
TNR	proteintech (19730-1-AP)	WB: 1/1000 IHC: 1/400
VAMP5	proteintech (11822-1-AP)	WB: 1/1000 IHC: 1/400
IGFBP2	BIOSS (bs-1108R)	WB: 1/1000 IHC: 1/400
TAGLN2	Santa (sc-373928)	WB: 1/1000 IHC: 1/400
METTL7B	Abclonal (A7200)	WB: 1/1000 IHC: 1/400
VIM	BIOSS (bs-8533R)	WB: 1/1000 IHC: 1/400

### Immunohistochemistry (IHC)

We selected 154 glioma chip tissues for IHC staining. The expressions of seven core genes and their relationships with patient survival were analyzed. For IHC, the experiments were performed as described previously (20). Antibodies used in the IHC staining are listed in **Table 2**. The IHC score of target proteins was independently evaluated by two pathologists according to the proportion and intensity of positive cells within five microscopic visual fields per slide (200-fold magnification) (21). A proportion score represented the estimated proportion of positively stained tumor cells and the intensity score represented the average intensity of the positive tumor cells. The proportion and intensity scores were then multiplied to obtain a total score, which ranged from 0 to 16. A total score of 0-2, 3-7, 8-12, 13-16 was defined as being negative (-), weak positive (+), moderate positive (++), and strong positive (+++), respectively (21).

### Flow Cytometry

We selected fresh tissues from glioma patients with grade II, III and IV. The tissues were cleaned with PBS and were cut into pieces with ophthalmic scissors. Then the tissues were collected into a 15ml centrifuge tube with 7ml PBS, then 1ml collagenase I was added into the centrifuge tube. Single cell suspension was collected with 700 mesh cell sieves. After washing by PBS, fluorescent antibodies CD3, CD4, CD8, CD11, CD16 and CD22 (listed in **Table 3**, purchased from Thermo Fisher Scientific) were added to cell suspension at a ratio of 1:20. After incubation at 4°C for 1h, they were cleaned by PBS for 3 times. The cell precipitates were resected with 500ul PBS and detected by flow cytometry.

**Table 3:** Primary antibodies used for Flow cytometry.

Antibody	Company (Cat. No.)	Working Dilutions
CD3-FITC	eBioscience (11-0037-42)	FCM: 1/20
CD4-PE	eBioscience (12-0049-42)	FCM: 1/20
CD8-PE	eBioscience (12-0088-42)	FCM: 1/20
CD11-PE	eBioscience (CD11B04-4)	FCM: 1/20
CD16-FITC	eBioscience (11-0168-42)	FCM: 1/20
CD22-PE	eBioscience (12-0029-41)	FCM: 1/20

## Results

### Identification of Three Immune-Subgroups Based on 25 Immune-Related Gene Sets

**Figure 1:**
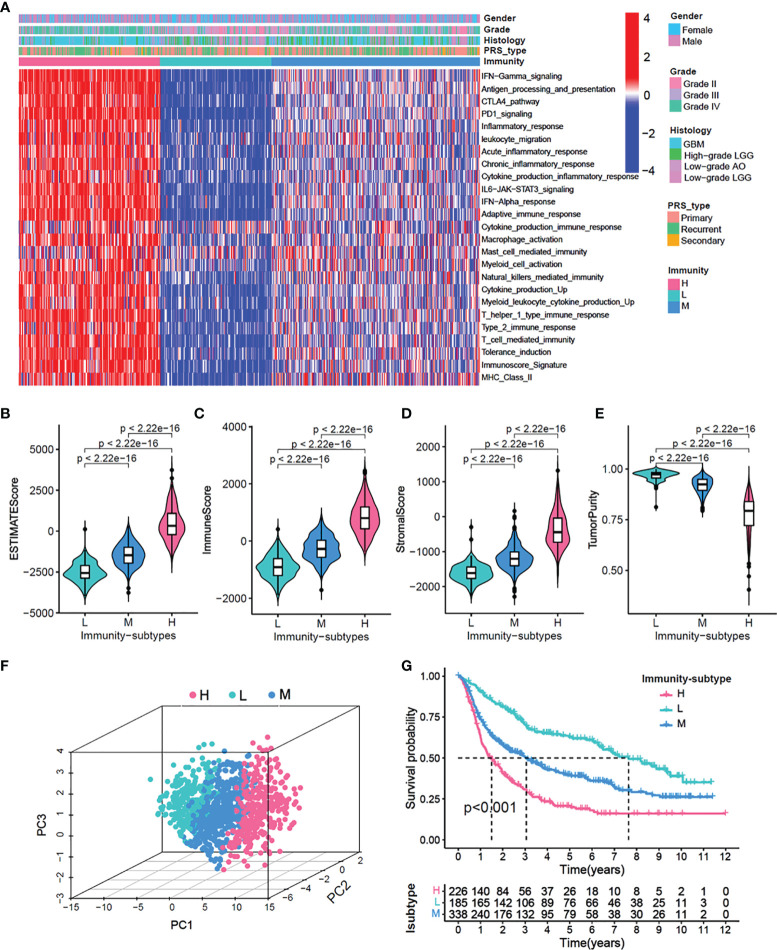
Identification of three immunity-subgroups base on 25 immunity-related gene sets. **(A)** The heatmap showing the glioma samples divided into three distinct sub-group based on the GSVA enrichment scores of 25 immunity-related gene sets. Three subgroups defined as immunity-H, immunity-M and immunity-L. Color bars at the top of the graph labels, the gender, grade, histology, PRS type. **(B–E)** Comparison of stromal scores **(B)**, immune scores **(C)**, estimated scores **(D)**, tumor purity **(E)** among three immunity-subgroups. **(F)** PCA analyses for three immunity-subgroups depicted by the dot in different colors. immunity-H, pink, immunity-M, blue; immunity-L, aquamarine. **(G)** OS Kaplan-Meier survival analysis among three immunity-subgroups (Log-rank test).

Using the CGGA cohort, first, we performed an unsupervised clustering analysis and identified three distinct immune-subgroup patterns based on the ssGSEA scores of 25 immune-related gene sets (**Figures 1A**, **S1A–E**). The three immune-subgroups were correspondingly termed as the immune-H (high) (n = 226), immune-M (media) (n = 185), and immune-L (low) (n=338). The immune-H subgroup had the highest values of ESTIMATE Score, Immune Score, and Stromal Score; it was also associated with the lowest tumor purity (**Figures 1B–E**). Notably, these results indicated that the immune-H subgroup consisted of the highest number of immune cells and stromal cells, while the immune-L subgroup consisted of the highest number of tumor cells; these values in the immune-M subgroup were between in those for the immune-H and immune-L subgroups. Moreover, the 3D-PCA plot showed that the immune subtypes were also distinctly divided into three clusters, namely the immune-H, immune-M, and immune-L, which suggested that our method could well distinguish among the three immune-subgroups (**Figure 1F**). Patients in the immune-L subgroup showed better overall survival, while those in the immune-H subgroup exhibited the worst prognosis (**Figure 1G**) (log-rank test, P < 0.001).

### Association Analysis and Risk Score Comparison of Clinical Features of Glioma

**Figure 2:**
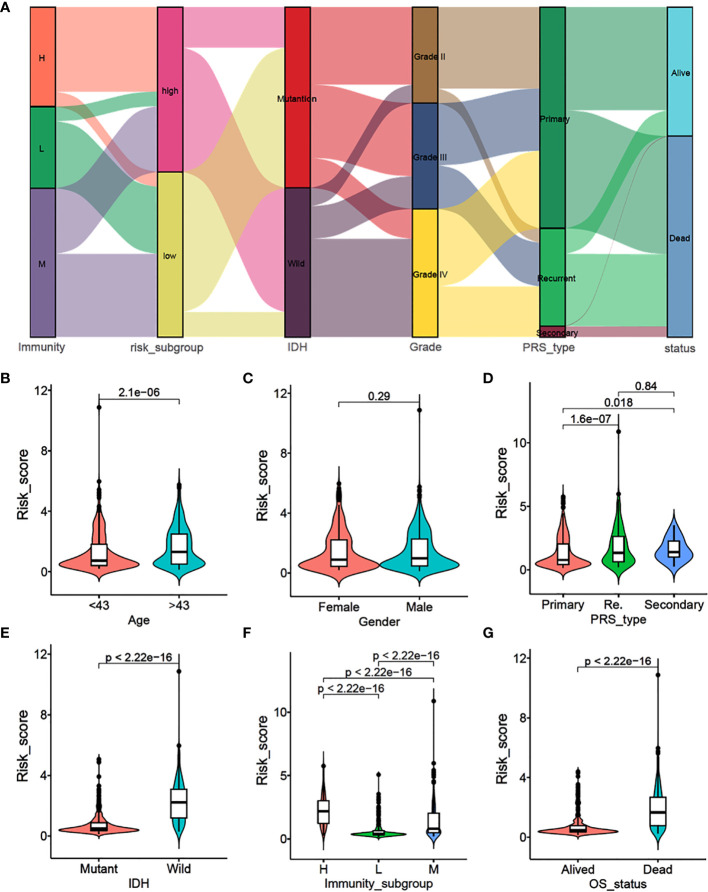
Association analysis and risk score comparison of clinical features of glioma. **(A)** Alluvial diagram of immunity-sub-groups with different risk-subgroups, IDH status, grade stages, PRS types and survival status. **(B–G)** The risk score comparison between different clinical features. **(B)** Age, **(C)** Gender, **(D)** PRS type, **(E)** IDH status, **(F)** immunity-subgroup and **(G)** survival status.

To explore the correlation between immune subtypes and clinical characteristics, we drew an alluvial diagram for immune-subgroups with different risk-subgroups, IDH status, grade stages, PRS types, and survival status (**Figure 2A**). The results indicated that the immune-H subgroup with the higher risk score was most likely related to IDH wild type, higher grades, recurrence status, and death. Whereas the immune-L subgroup exhibited a lower risk score, IDH mutation, lower grade, primary status, and better survival status. The correlation results are specifically shown in **Figures S2A–F** and **S3A–F**. Statistical analysis of the clinical data showed that higher risk scores were more likely associated with older age, higher recurrence, IDH wild type, higher immune subtype, and poorer prognosis (*P*<0.001), while there were no significant differences between gender (**Figures 2B–G**). At the same time, we divided gliomas into low grade and high grade groups to analyze the relationship between different immune subtypes and patient prognosis. We found that high immune subtypes were significantly correlated with poor prognosis of patients with low grade (Left) and high grade (Right) glioma patients (**Figure S2G**).

### Enriched Molecular Pathways and Immune Cell Infiltration Among Three Immune-Subgroups of Glioma

**Figure 3:**
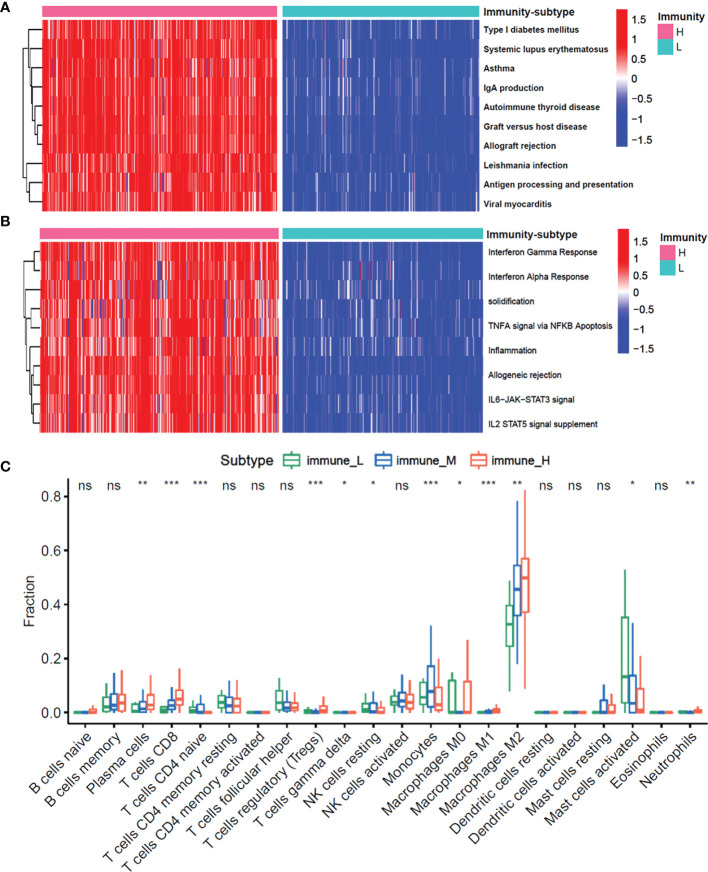
The enrichment molecular pathways and immune cells infiltration among three immunity-subgroups of glioma. **(A, B)** Heatmap shows the GSVA score of top 10 KEGG pathways **(A)** and 50 hallmarks pathways **(B)** curated from MSigDB between immunity-H and immunity-L subtypes. **(C)** The abundance of each 22 types of infiltrating cell in three immunity-subtypes. *P≤0.05, **P≤0.01, ***P≤0.001. ns, no significance.

To examine the molecular mechanisms of action in different subtypes, we performed the gene set variation analysis (GSVA) to evaluate the enriched pathways in the KEGG and hallmark gene sets. **Figures 3A, B** shows the differentially enriched pathways from the KEGG database and hallmark gene sets between the immune-H and immune-L subgroups. Enriched pathways in the immune-H subgroup mainly comprised immune-related pathways, including systemic lupus erythematosus, inflammation, and antigen processing and presentation. Similar results were obtained in immune-H vs immune-M and immune-M vs immune-L subgroup analyses (**Figures S4A–D**).

**Figure 4:**
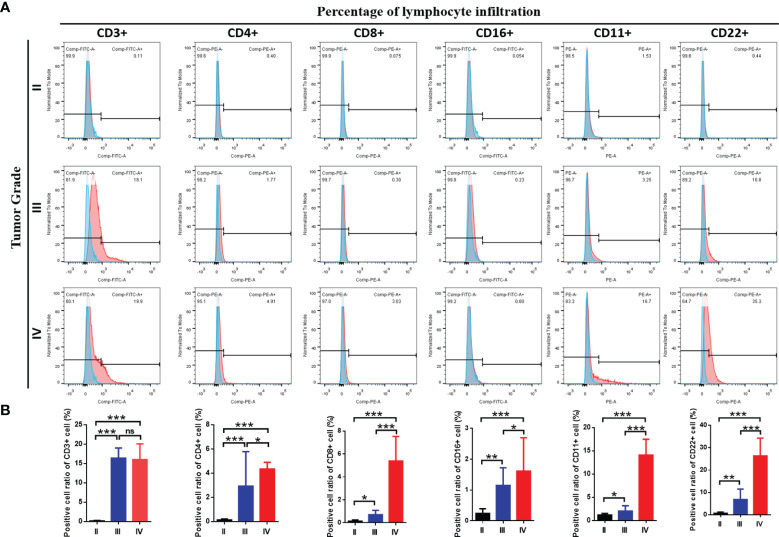
The immune cells infiltration among three grades of glioma. **(A)** Flow cytometry results showed the abundance of each 6 types of infiltrating cell in three grades and **(B) **the results of statistical analysis showed the proportion of cell infiltration.*P≤0.05, **P≤0.01, ***P≤0.001. ns, no significance.

Moreover, we investigated whether the three immune-subgroups of glioma had different tumor immune microenvironments (TIME) (**Figure 3C**). Indeed, the immune-H subgroup had high infiltration levels of regulatory T cells (Tregs), M2 macrophages, and neutrophils, while the immune-L subgroup had remarkable enrichment of resting mast cells. Furthermore, we found a significant increase of CD3, CD4, CD8, CD16, CD11, CD22 positive immune cell infiltrates in grade IV glioma tissues compared with grade II and III (**Figures 4A, B**). These results indicated that the gliomas in the immune-H subgroup and grade IV glioma group were dominated by infiltration of suppressive immune cell types.

To test the expression of immune-related genes in each group, we examined the expression of HLA and the immune checkpoint genes in the three immune subgroups. Notably, the expressions of all the HLA genes were highest in the immune-H subgroup, while lowest in the immune-L subgroup (ANOVA test, *P* < 0.001) (**Figure S5A**). Further analysis of immune checkpoints showed that multiple inhibitory checkpoints (CD274, CTLA4, HAVCR2, LAG3, PDCD1LG2, TIGIT) were significantly overexpressed in high immune subtypes (**Figure S5B**). These results showed that the immune-subgroups were significantly associated with the expressions of immune-related genes.

### Differential Gene Expression Analysis Among Three Immune-Subgroups

**Figure 5:**
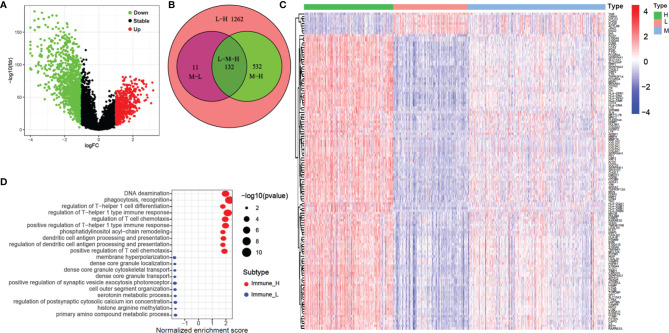
Volcano plot, heatmap and GO enrichment analysis of differential gene among three glioma immune subtype. **(A)** The volcano plot of different genes between immunity-H and immunity-L subtype. **(B) **The Venn plot showed the different genes among three glioma immune subtype. **(C) **The heatmap shown that the different genes among three glioma immune subtype. **(D)** GO enrichment analysis of differential gene among between immunity-H and immunity-L subtypes.

We identified a total of 1937 DEGs (|log_2_FC| > 1 and FDR < 0.05) between the immune-H and immune-L subgroups and the volcano plot was used to show the distribution of the DEGs between the two subgroups (**Figure 5A**). Specifically, 1262 DEGs in immune-H vs immune-L, 532 DEGs in immune-H vs immune-M, 11 DEGs in immune-M vs immune-L, and 132 DEGs in immune-H vs immune-M vs immune-L were obtained (**Figure 5B**). As shown in **Figure 5C**, the heatmap displays 132 DEGs among the three groups. The GSEA for GO enrichment showed that the immune-H subgroup was highly enriched in genes associated with the immune-related pathways (**Figure 5D**), including immunoglobulin complex, immunoglobulin receptor binding, complement activation, T cell receptor complex, and immune response-regulating cell surface receptor signaling pathway involved in phagocytosis. This result was in line with elevated immune activity in the immune-H group.

### Construction of the Prognostic Model Based on Glioma Immunophenotyping

**Figure 6:**
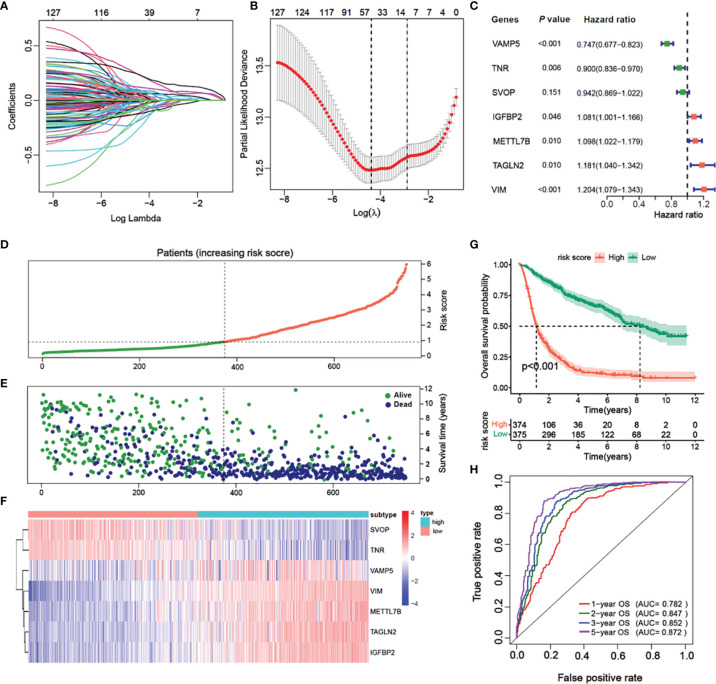
Construction of a prognostic model based on differential genes of immune subtypes. **(A)** The LASSO coefficients profile of the 132 significate genes selected for overall survival against the log lambda sequence. **(B)** Ten-fold cross-validated error (first vertical line equals the minimum error, whereas the second vertical line shows the cross-validated error within 1 standard error of the minimum). **(C)** Forest plot of 7 immunity-subtype-related genes identified by multivariate Cox regression. **(D)** Glioma patients are sorted by risk score, red is high risk, green is low risk. **(E)** The survival status of Glioma patients, dark blue is dead, light green is alive. **(F)** The heatmap of the 7-hub gene expression. **(G)** Kaplan–Meier curve survival analysis between high risk and low risk, red line means high risk group, blue line means low risk group; **(H)** Time–ROC curve analysis of the 7 hub genes signature, red line means 1-year OS, green line means 2-year OS, blue line means 3-year DFS, purple line means 3-year DFS.

In the CGGA cohort, 14 of 132 DEGs were identified and selected through the lasso-Cox regression algorithm (**Figures 6A, B**). Finally, a total of seven prognostic-related hub genes were identified by multivariate Cox regression analysis, and the risk scores were calculated to predict the prognostic risk of glioma (**Figures 6C–F**). The multivariate Cox forest plot showed the hazard ratios of SVOP, TNR, VAMP5, IGFBP2, METTL7B, VIM, and TAGLN2. The risk score was calculated using the following formula: risk score $=\sum_{i}^{n}coef_{i}\;*\;mRNA_{i}$ , where i, represents the expressions of the seven hub genes. The risk score, survival status, and the expressions of the seven hub genes were calculated using the prognostic model and the process is illustrated in **Figures 6D–F**. Samples were classified into low- and high-risk subgroups according to the median risk score. Survival analysis indicated that patients in the low-risk subgroup had significantly longer overall survival time as compared to that of the high-risk patients (**Figure 6G**, P < 0.001, log-rank test). ROC curve analysis showed that the specificity and sensitivity were highest when the risk scores were 0.782, 0.843, 0.852, and 0.857 according to the 1-, 2-, 3-, and 5-year survival-based area under the receiver operating characteristic curve (AUC) values, respectively (**Figure 6H**).

### Univariate Cox and K-M Survival Analysis of the Seven Hub Genes in CGGA and TCGA Cohorts

**Figure 7:**
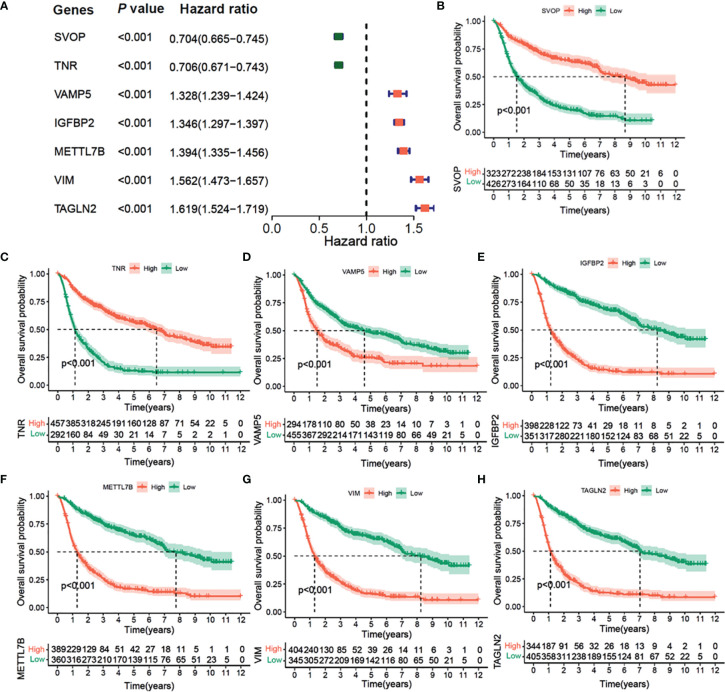
Univariate Cox Forest plot and Kaplan–Meier survival analysis of 7 hub genes in CGGA cohort. **(A)** Univariate Cox Forest plot of 7 immunity-subtype-related genes. **(B)** Kaplan–Meier curve survival analysis of 7 hub genes, **(B)** SVOP, **(C)** TNR, **(D)** VAMP5, **(E)** IGFBP2, **(F)** METTL7N, **(G)** VIM, **(H)** TAGLN2.

The results of univariate Cox regression analysis of the seven prognostic-related genes are shown in **Figure 7A** in CGGA cohort. Among them, SVOP and TNR were determined as the protective factors with HR values*<*1, whereas the remaining five genes, VAMP5, IGFBP2, METTL7B, VIM, and TAGLN2 were determined as the risk factors with hazard ratio (HR) values >1. According to the optimal cutoff value, a total of 749 samples were divided into two groups according to the expression levels of the seven genes. Survival analysis indicated that patients with high expression of SVOP or TNR had significantly longer overall survival than those with low expression of SVOP/TNR (**Figures 7B, C**), while the results of VAMP5, IGFBP2, METTL7B, VIM, and TAGLN2 showed opposite trends (**Figures 7D–H**). Similar results were obtained in TCGA cohort (**Figures S4A–H**).

Furthermore, we compared the expressions of the seven hub genes among GBM, LGG, and normal tissues. The expression of SVOP was upregulated in the normal tissue than in the GBM (GBM < LGG < normal tissues) (**Figures S7A**). The expressions of VAMP5, IGFBP2, METTL7B, VIM, and TAGLN2 were lower in normal tissue than in GBM (GBM > LGG > normal tissues) (**Figures S7C–G**), while the expressions of TNR in LGG was the highest as compared to both the groups (**Figures S7B**). Subsequently, the glioma patients were divided into low grade and high grade in CCGA and TCGA databases respectively, and the relationships between seven core genes and prognosis of patients were analyzed (**Figures S8A–D**). The results were basically consistent with **Figures 6** and **S7**.

### The Seven Hub Genes Are Significantly Related to Immune Cell Infiltration and T-Cell Inflammation-Associated GEP

**Figure 8:**
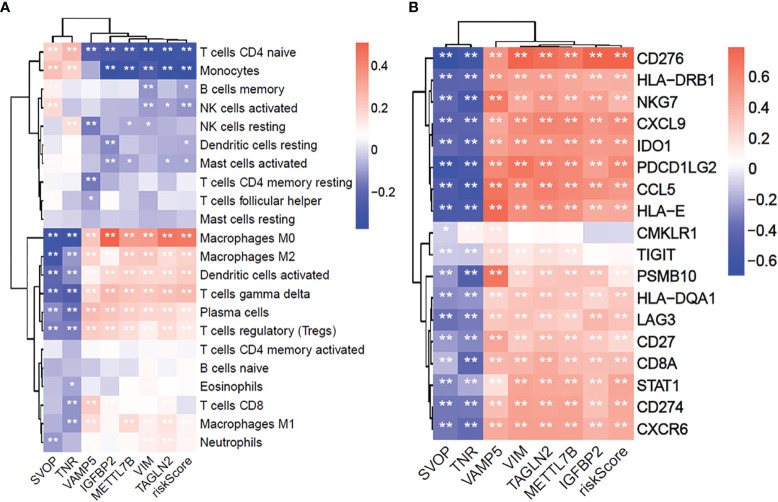
The 7 core prognostic genes of glioma are significantly related to immune cells infiltration and GEP genes. **(A)** The correlation between 7 prognostic hub genes and 22 immune cells infiltration. **(B)** The correlation between 7 prognostic hub genes and 18 GEP expression. *P≤0.05, **P≤0.01.

To examine the correlation between the seven prognostic-related genes and immune cell infiltrations, the relative abundances of 22 types of infiltrating immune cells in gliomas were quantified using CIBERSORT (**Figure 8A**). The SVOP and TNR expression had a negative correlation with regulatory T cells (Tregs), M2 macrophages, and neutrophils, while the risk scores based on VAMP5, IGFBP2, METTL7B, VIM, TAGLN2 were significantly positively correlated with these immunosuppressive cell types.

Furthermore, we explored the correlation between the seven hub genes and T-cell inflammation-associated gene expression profile (GEP) (16). The results showed that the SVOP and TNR expressions were negatively associated with T-cell inflammation GEP, while VAMP5, IGFBP2, METTL7B, VIM, TAGLN2 were significantly positively associated with T-cell inflammation-related GEP (**Figure 8B**). These results confirmed that the seven hub genes were significantly related to the immune response and may be potential markers for predicting immunotherapeutic responses.

### Functional Validation of the Seven Hub Genes in Clinical Specimens

**Figure 9:**
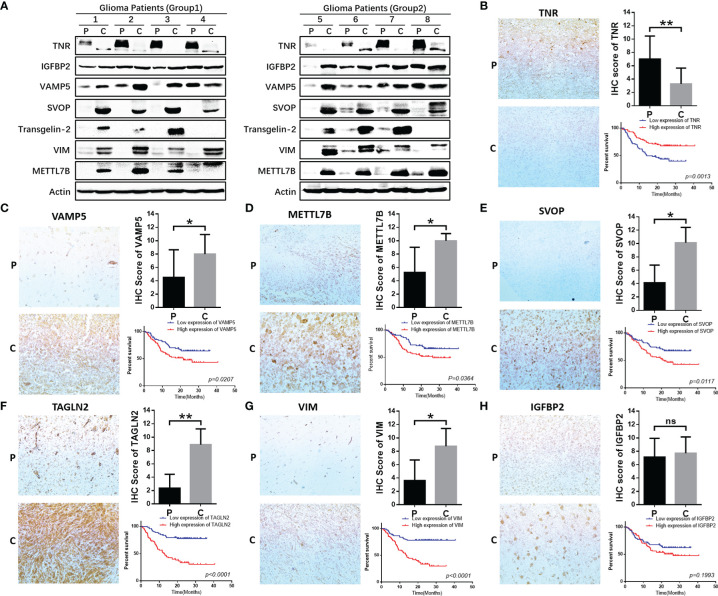
Functional validation of 7 hub genes in clinical specimens. **(A)** WB assay was used to detect the expression differences of 7 core genes in glioma tissues and adjacent tissues. **(B–H)** Immunohistochemical staining and statistical analysis of tumor and adjacent tissues in patients with glioma, and Kaplan-Meier curve analysis of overall survival in glioma patients by the expression of 7 core genes. Death/total number of patients in each subgroup were presented. (P, paracancerous; C, cancer). *P≤0.05, **P≤0.01, ***P≤0.001. ns, no significance.

We verified the expression of seven core molecules at the tissue levels, and the results showed that the expression of five molecules (VAMP, SVOP, Transgelin2, VIM, METTL7B) in glioma tissues was significantly higher than that in adjacent tissues, while the expression of TNR was on the contrary, and IGFBP2 had no significant significance (**Figure 9A**).

We further validated the protein expression levels of these genes in clinical specimens. A total of 154 glioma samples were divided into high-expression (n=77) and low-expression (n=77) groups according to the IHC scores for the seven genes. Five of these seven genes were significantly related to the survival of glioma patients and one among them did not show statistical significance (**Figures 9B–H**). However, the expression and prognosis of SVOP were contrary to the results from the database analysis. Further investigation of the function and mechanism of these molecules is needed.

## Discussion

The WHO classification guidelines for central nervous system tumors (2021 Edition) classify several tumors with specific molecular changes into unique subgroups. This is of great significance to obtain different prognostic results and can guide the formulation of individualized treatment strategies (2). With the deepened understanding of the immune system, several immunotherapeutic agents have been tested in clinical trials, but most of them ended in failure. One of the underlying reasons is that these clinical trials did not fully consider the characteristics of the patients’ tumor immune microenvironment and failed to distinguish and identify suitable enrolled patients (8). The tumor immune microenvironment is extremely complex, which not only includes a large number of highly heterogeneous tumor cells, but also different types of immune cells and states such as exhaustion and decrepit, thereby leading to mixed effects in immunotherapeutic responses. The current understanding of the glioma immune microenvironment is insufficient to guide clinical practice.

Immunotherapy has revolutionized tumor therapy. In this therapeutic strategy, the drugs can penetrate the blood-brain barrier for the treatment of glioma (22). However, many clinical trials for immunotherapy have not shown favorable results (8, 9). The main reason may be attributed to the complex tumor immune microenvironment, leading to significant heterogeneity in the tumor microenvironment of different patients; thus, the individualized treatment was not easy to achieve (23, 24). Given this, we screened the genetic data in CGGA and TCGA databases and explored the data in the perspective of glioma immune microenvironment classification. A total of seven immune genes related to glioma prognosis were identified and protein expression verification based on pathological tissue samples was performed. In this study, we divided the glioma patients in CGGA and TCGA cohort into three immune subtypes according to their GSVA enrichment scores. The immune-H subtype was significantly correlated with the poor prognosis in all glioma patients. This suggested that our immune-typing may have direct significance for immunotherapy of glioma patients.

Although glioma immunotherapy has been unfavorable, studies on the glioma immune microenvironment have attracted great attention. Previous studies based on single-cell sequencing show significant heterogeneity in tumors (25, 26). This is also one of the possible reasons for the complexity of the tumor immune microenvironment. Therefore, it is important to examine the underlying molecular mechanisms for different immune subtypes in different tumor types. In this study, we queried the KEGG and hallmarks pathways and found that differentially enriched pathways showing significant differences between the immune-H and immune-L subtypes were mainly related to the regulation of the immune microenvironment in glioma. In the immune-H subtype, the immune cells showed higher infiltration than those in the immune-M and immune-L subtypes. Furthermore, 132 genes with significant differential expression were screened by GO enrichment and were found to be mainly enriched in immune-related pathways. Seven immune-related core genes were further screened by LASSO regression analysis and the correlation with the prognostic risk of patients was found to be significant. Through prognostic analysis, these seven genes were found to be significantly correlated with the prognosis of glioma patients and the high-risk score subgroup was significantly correlated with the poor prognosis of these patients. Thus, these identified genes may be potential new targets for immunotherapy for glioma patients and may be of great importance for screening and identifying patients who could benefit from immunotherapy.

Search for new immunotherapeutic strategies is critical to overcoming the obstacles in glioma immunotherapy, however, the molecular markers identified in many previous studies couldn’t be transformed for clinical applicability (27). At present, there were few reports on the 7 molecules we screened, the mechanism involved with tumor immune microenvironment is not clear. Bo-Ra Na have shown that TAGLN 2 is the only molecule in TAGLN family that is related to immune cells and participated in the regulation of immune cell activity (28). Hye-Ran Kim have shown that TAGLN2 could participate in lipopolysaccharide induced macrophage activation through NF KB pathway (29). Through database analysis, some scholars found that mettl7b was highly expressed in gliomas and were associated with poor prognosis, but its specific molecular mechanism was not clear (30). Ting Li have pointed out that IGFBP2 can regulate PD-L1 expression by activating EGFR-STAT3 signaling pathway in melanoma and participate in tumor immunotherapy resistance, but its specific mechanism needs to be further studied (31). However, the mechanism of other molecules in the regulation of tumor immune microenvironment have not been reported.

In our study, the expression of seven core genes in tumor and para tumor tissues was verified by immunohistochemistry and western blotting. The results showed that the expressions of five genes (TNR, VAMP5, METTL7B, TAGLN2, VIM) were high and consistent with the findings from the CGGA and TCGA databases. However, the expression and prognostic prediction for SVOP were contrary to the database findings and there were no significant differences in the expression and prognosis of IGFBP2.

Through analyzing the TCGA-GBM and CGGA-GBM data, the results of SVOP molecules in different databases are quite opposite, and our validation data correlated with that in TCGA database. We believe that the possible reasons include that there are still defects in the current understanding of molecular typing of glioma patients, and the possible effects of IDH, P53, 1P/19Q, PTEN, TERT promoter methylation status and MGMT promoter methylation status on the prognosis of patients are not fully considered in the analysis. According to the report of Prof. Zhao et al. (32), in TCGA database, SVOP is related to hypermethylation status and prognosis in patients with GBM, which may be one of the reasons for inconsistent conclusions, but the internal mechanism has not been reported. SVOP and IGFBP2 may play different functions in different grades of gliomas and follow-up studies in these contexts are in progress.

In conclusion, we analyzed the CGGA and TCGA databases for immune functions, grouped glioma patients, and investigated the specificity and complexity of the glioma immune microenvironment. The identified seven core genes may provide potential targets for glioma immunotherapy and were significantly related to the prognosis of glioma patients. These results emphasize the importance of tumor immunotyping in the development of glioma. Therefore, targeting the related molecules for immunoassay is expected to become a feasible treatment strategy in combined immunotherapy.

## Data Availability Statement

Publicly available datasets were analyzed in this study. This data can be found here: CGGA databases: http://www.cgga.org.cn/; TCGA databases: https://cancergenome.nih.gov/.

## Ethics Statement

The studies involving human participants were reviewed and approved by IEC of Institution for National Drug Clinical Trials, Tangdu Hospital, Fourth Military Medical University. Written informed consent to participate in this study was provided by the participants’ legal guardian/next of kin.

## Author Contributions

X-XW, HC, and YZ designed research, analyzed and interpreted data, and wrote the manuscript. S-ZD, MC, YH, YM, and SG performed research and collected, analyzed, and interpreted data. WZ, CL, and YJ collected data, performed IHC, data analysis and manuscript preparation. X-XW performed bioinformatics, data analysis and manuscript preparation. GX and LH performed research. H-MZ and LW designed research, guided experiment and wrote the manuscript. Author order reflects the relative size and importance of the contributions to the project and manuscript. All authors contributed to the article and approved the submitted version.

## Funding

This work was supported by the National Natural Science Foundation of China (nos. 81802772, 81772661), the Natural Science Foundation of Shaanxi Province (nos. 2020JZ-30, 2021JZ-35), Clinical Study of Toripalimab with Anlotinib for Patients with Recurrent Glioblastoma (nos. ChiCTR2000039175), Talent Project of Tangdu Hospital, The Fourth Military Medical University (nos. 2021ZTXM016, 2021ZTXM007, 2021SHRC021, 2021SHRC020, 2021SHRC033, 2021SHRC001).

## Conflict of Interest

The authors declare that the research was conducted in the absence of any commercial or financial relationships that could be construed as a potential conflict of interest.

## Publisher’s Note

All claims expressed in this article are solely those of the authors and do not necessarily represent those of their affiliated organizations, or those of the publisher, the editors and the reviewers. Any product that may be evaluated in this article, or claim that may be made by its manufacturer, is not guaranteed or endorsed by the publisher.

## References

[1] Ilkhanizadeh S Lau J Huang M Foster DJ Wong R Frantz A. Glial Progenitors as Targets for Transformation in Glioma. Adv Cancer Res (2014) 121:1–65. doi: 10.1016/B978-0-12-800249-0.00001-9 24889528 PMC4270964

[2] Yuan L Shen J Chen Z. Catalytic Ozonation of P-Chloronitrobenzene Over Pumice-Supported Zinc Oxyhydroxide. Water Sci Technol (2013) 68(8):1895–900. doi: 10.2166/wst.2013.449 24185076

[3] Stupp R Brada M van den Bent MJ Tonn JC Pentheroudakis G. High-Grade Glioma: ESMO Clinical Practice Guidelines for Diagnosis, Treatment and Follow-Up. Ann Oncol (2014) 25(Suppl 3):iii93–101. doi: 10.1093/annonc/mdu050 24782454

[4] Tan AC Ashley DM Lopez GY Malinzak M Friedman HS Khasraw M. Management of Glioblastoma: State of the Art and Future Directions. CA Cancer J Clin (2020) 70(4):299–312. doi: 10.3322/caac.21613 32478924

[5] Tong N He Z Ma Y Wang Z Huang Z Cao H. Tumor Associated Macrophages, as the Dominant Immune Cells, Are an Indispensable Target for Immunologically Cold Tumor-Glioma Therapy? Front Cell Dev Biol (2021) 9:706286. doi: 10.3389/fcell.2021.706286 34368156 PMC8337013

[6] Xie Y He L Lugano R Zhang Y Cao H He Q. Key Molecular Alterations in Endothelial Cells in Human Glioblastoma Uncovered Through Single-Cell RNA Sequencing. JCI Insight (2021) 6(15):150861. doi: 10.1172/jci.insight.150861 PMC8410070 34228647

[7] Reardon DA Brandes AA Omuro A Mulholland P Lim M Wick A. Effect of Nivolumab vs Bevacizumab in Patients With Recurrent Glioblastoma: The CheckMate 143 Phase 3 Randomized Clinical Trial. JAMA Oncol (2020) 6(7):1003–10. doi: 10.1001/jamaoncol.2020.1024 PMC7243167 32437507

[8] Buerki RA Chheda ZS Okada H. Immunotherapy of Primary Brain Tumors: Facts and Hopes. Clin Cancer Res (2018) 24(21):5198–205. doi: 10.1158/1078-0432.CCR-17-2769 PMC6214775 29871908

[9] Mangani D Weller M Roth P. The Network of Immunosuppressive Pathways in Glioblastoma. Biochem Pharmacol (2017) 130:1–9. doi: 10.1016/j.bcp.2016.12.011 28017775

[10] Martinez-Lage M Lynch TM Bi Y Cocito C Way GP Pal S. Immune Landscapes Associated With Different Glioblastoma Molecular Subtypes. Acta Neuropathol Commun (2019) 7(1):203. doi: 10.1186/s40478-019-0803-6 31815646 PMC6902522

[11] Simpson KD Templeton DJ Cross JV. Macrophage Migration Inhibitory Factor Promotes Tumor Growth and Metastasis by Inducing Myeloid-Derived Suppressor Cells in the Tumor Microenvironment. J Immunol (2012) 189(12):5533–40. doi: 10.4049/jimmunol.1201161 PMC3518629 23125418

[12] Gieryng A Pszczolkowska D Walentynowicz KA Rajan WD Kaminska B. Immune Microenvironment of Gliomas. Lab Invest (2017) 97(5):498–518. doi: 10.1038/labinvest.2017.19 28287634

[13] Quail DF Joyce JA. Microenvironmental Regulation of Tumor Progression and Metastasis. Nat Med (2013) 19(11):1423–37. doi: 10.1038/nm.3394 PMC3954707 24202395

[14] Shi Y Ping YF Zhou W He ZC Chen C Bian BS. Tumour-Associated Macrophages Secrete Pleiotrophin to Promote PTPRZ1 Signalling in Glioblastoma Stem Cells for Tumour Growth. Nat Commun (2017) 8:15080. doi: 10.1038/ncomms15080 28569747 PMC5461490

[15] Friebel E Kapolou K Unger S Nunez NG Utz S Rushing EJ. Single-Cell Mapping of Human Brain Cancer Reveals Tumor-Specific Instruction of Tissue-Invading Leukocytes. Cell (2020) 181(7):1626–42.e20. doi: 10.1016/j.cell.2020.04.055 32470397

[16] Cristescu R Mogg R Ayers M Albright A Murphy E Yearley J. Pan-Tumor Genomic Biomarkers for PD-1 Checkpoint Blockade-Based Immunotherapy. Science (2018) 362(6411):3593. doi: 10.1126/science.aar3593 PMC6718162 30309915

[17] Wilkerson MD Hayes DN. ConsensusClusterPlus: A Class Discovery Tool With Confidence Assessments and Item Tracking. Bioinformatics (2010) 26(12):1572–3. doi: 10.1093/bioinformatics/btq170 PMC2881355 20427518

[18] Hanzelmann S Castelo R Guinney J. GSVA: Gene Set Variation Analysis for Microarray and RNA-Seq Data. BMC Bioinf (2013) 14:7. doi: 10.1186/1471-2105-14-7 PMC3618321 23323831

[19] Chen B Khodadoust MS Liu CL Newman AM Alizadeh AA. Profiling Tumor Infiltrating Immune Cells With CIBERSORT. Methods Mol Biol (2018) 1711:243–59. doi: 10.1007/978-1-4939-7493-1_12 PMC5895181 29344893

[20] Huang Q Zhan L Cao H Li J Lyu Y Guo X. Increased Mitochondrial Fission Promotes Autophagy and Hepatocellular Carcinoma Cell Survival Through the ROS-Modulated Coordinated Regulation of the NFKB and TP53 Pathways. Autophagy (2016) 12(6):999–1014. doi: 10.1080/15548627.2016.1166318 27124102 PMC4922447

[21] Li J Huang Q Long X Zhang J Huang X Aa J. CD147 Reprograms Fatty Acid Metabolism in Hepatocellular Carcinoma Cells Through Akt/mTOR/SREBP1c and P38/PPARalpha Pathways. J Hepatol (2015) 63(6):1378–89. doi: 10.1016/j.jhep.2015.07.039 26282231

[22] Zeng J See AP Phallen J Jackson CM Belcaid Z Ruzevick J. Anti-PD-1 Blockade and Stereotactic Radiation Produce Long-Term Survival in Mice With Intracranial Gliomas. Int J Radiat Oncol Biol Phys (2013) 86(2):343–9. doi: 10.1016/j.ijrobp.2012.12.025 PMC3963403 23462419

[23] Chen Z Hambardzumyan D. Immune Microenvironment in Glioblastoma Subtypes. Front Immunol (2018) 9:1004. doi: 10.3389/fimmu.2018.01004 29867979 PMC5951930

[24] Xu S Tang L Li X Fan F Liu Z. Immunotherapy for Glioma: Current Management and Future Application. Cancer Lett (2020) 476:1–12. doi: 10.1016/j.canlet.2020.02.002 32044356

[25] Patel AP Tirosh I Trombetta JJ Shalek AK Gillespie SM Wakimoto H. Single-Cell RNA-Seq Highlights Intratumoral Heterogeneity in Primary Glioblastoma. Science (2014) 344(6190):1396–401. doi: 10.1126/science.1254257 PMC4123637 24925914

[26] Sottoriva A Spiteri I Piccirillo SG Touloumis A Collins VP Marioni JC. Intratumor Heterogeneity in Human Glioblastoma Reflects Cancer Evolutionary Dynamics. Proc Natl Acad Sci USA (2013) 110(10):4009–14. doi: 10.1073/pnas.1219747110 PMC3593922 23412337

[27] Griveau A Seano G Shelton SJ Kupp R Jahangiri A Obernier K. A Glial Signature and Wnt7 Signaling Regulate Glioma-Vascular Interactions and Tumor Microenvironment. Cancer Cell (2018) 33(5):874–89.e7. doi: 10.1016/j.ccell.2018.03.020 29681511 PMC6211172

[28] Na BR Kim HR Piragyte I Oh HM Kwon MS Akber U. TAGLN2 Regulates T Cell Activation by Stabilizing the Actin Cytoskeleton at the Immunological Synapse. J Cell Biol (2015) 209(1):143–62. doi: 10.1083/jcb.201407130 PMC4395477 25869671

[29] Kim HR Lee HS Lee KS Jung ID Kwon MS Kim CH. An Essential Role for TAGLN2 in Phagocytosis of Lipopolysaccharide-Activated Macrophages. Sci Rep (2017) 7(1):8731. doi: 10.1038/s41598-017-09144-x 28821818 PMC5562783

[30] Xiong Y Li M Bai J Sheng Y Zhang Y. High Level of METTL7B Indicates Poor Prognosis of Patients and Is Related to Immunity in Glioma. Front Oncol (2021) 11:650534. doi: 10.3389/fonc.2021.650534 33996568 PMC8117938

[31] Li T Zhang C Zhao G Zhang X Hao M Hassan S. IGFBP2 Regulates PD-L1 Expression by Activating the EGFR-STAT3 Signaling Pathway in Malignant Melanoma. Cancer Lett (2020) 477:19–30. doi: 10.1016/j.canlet.2020.02.036 32120023 PMC7816098

[32] Zhao J Wang L Kong D Hu G Wei B. Construction of Novel DNA Methylation-Based Prognostic Model to Predict Survival in Glioblastoma. J Comput Biol (2020) 27(5):718–28. doi: 10.1089/cmb.2019.0125 31460783

