# Supplementary material for: Immune Gene Signatures and Immunotypes in Immune Microenvironment Are Associated With Glioma Prognose
Source: Front Immunol. 2022 Apr 14;13:823910. doi: 10.3389/fimmu.2022.823910 (PMC9046586; doi:10.3389/fimmu.2022.823910)
Supplement: Supplementary file 1 [file DataSheet_1.pdf]

Supplementary materials

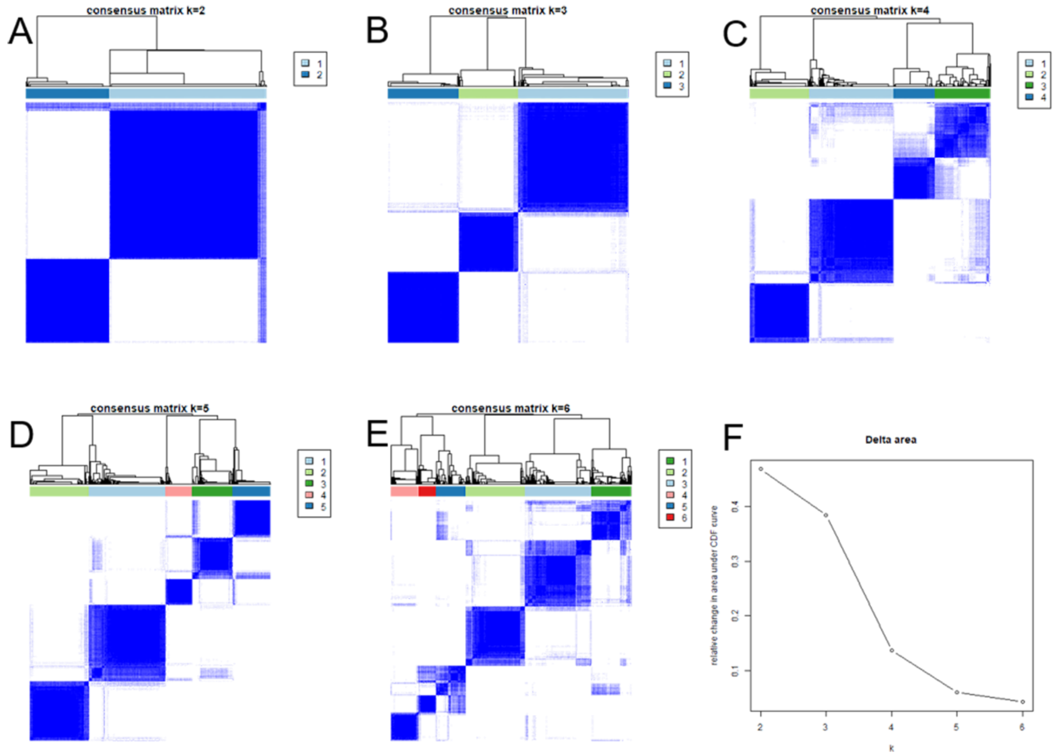

Figure s1. Unsupervised clustering of glioma samples base on 25 immunity-related gene sets. (A-E) Consensus matrices of CGGA cohort for k = 2-6. (F). Scree plot of consensus matrices of the merged CGGA cohort for k = 2-6.

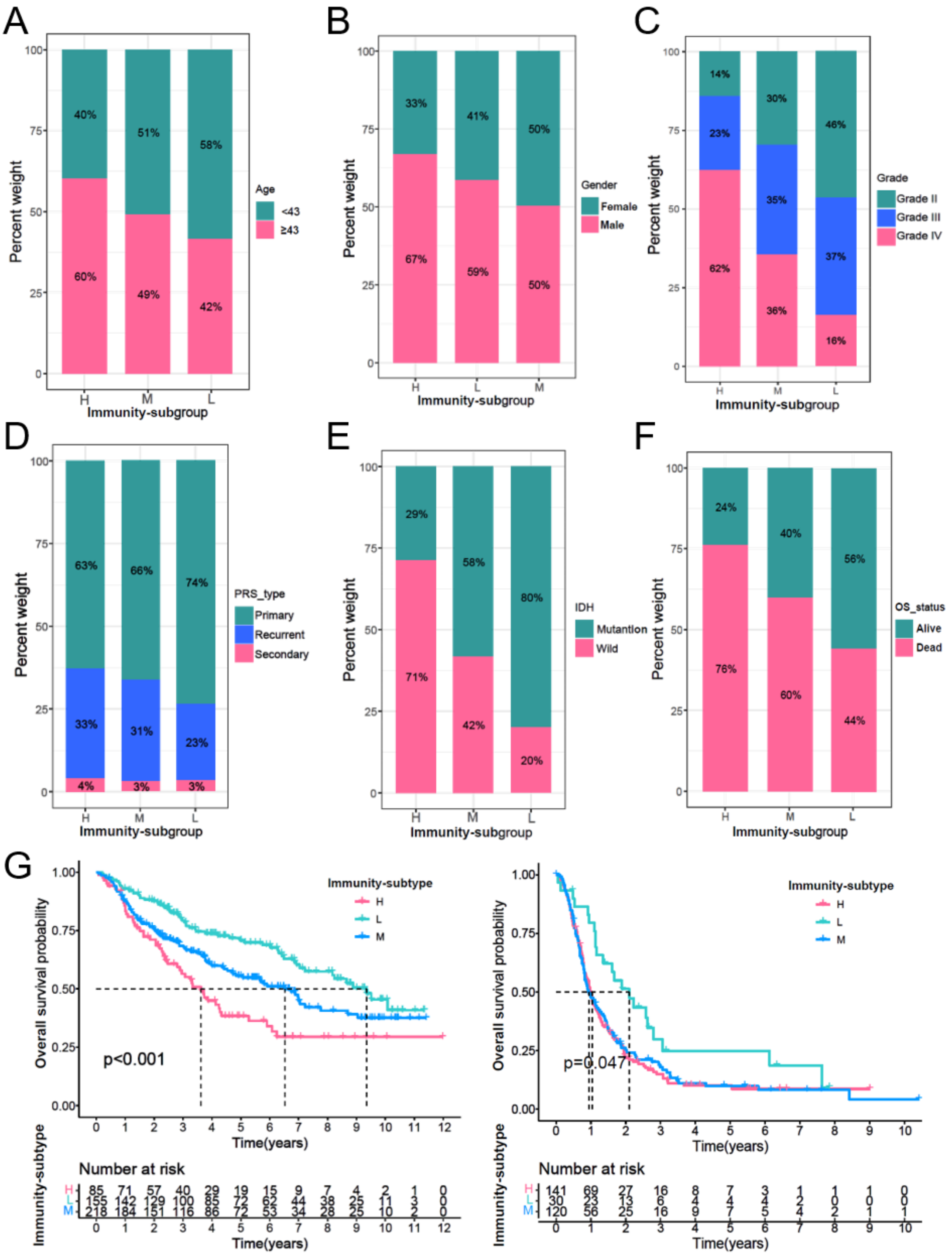

Figure S2. Distribution of clinical features among different immunity-subtypes of glioma. (A-F) Distribution of clinical features among different immune subtypes of glioma. (A) Age, (B) Gender, (C) survival status, (D) PRS type, (E) IDH status and (F) risk-subgroup. (G) Prognosis and survival analysis of different immune subtypes of glioma in PATIENTS with LGG (Left) or HGG (Right).

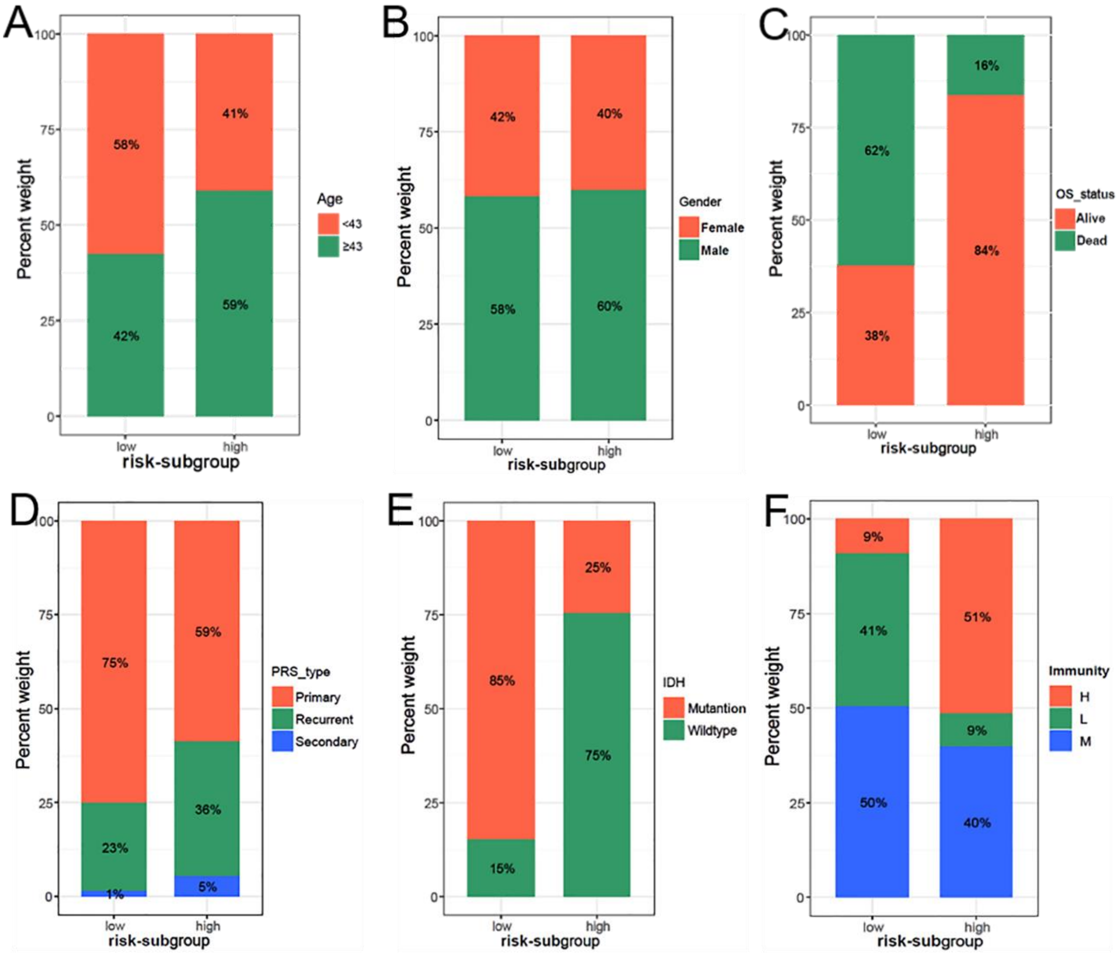

Figure S3. Distribution of clinical features among different risk-subgroups of glioma. (A-F) Distribution of clinical features among different risk-subgroups of glioma. (A) Age, (B) Gender, (C) survival status, (D) PRS type, (E) IDH status and (F) Immunity-subgroup.

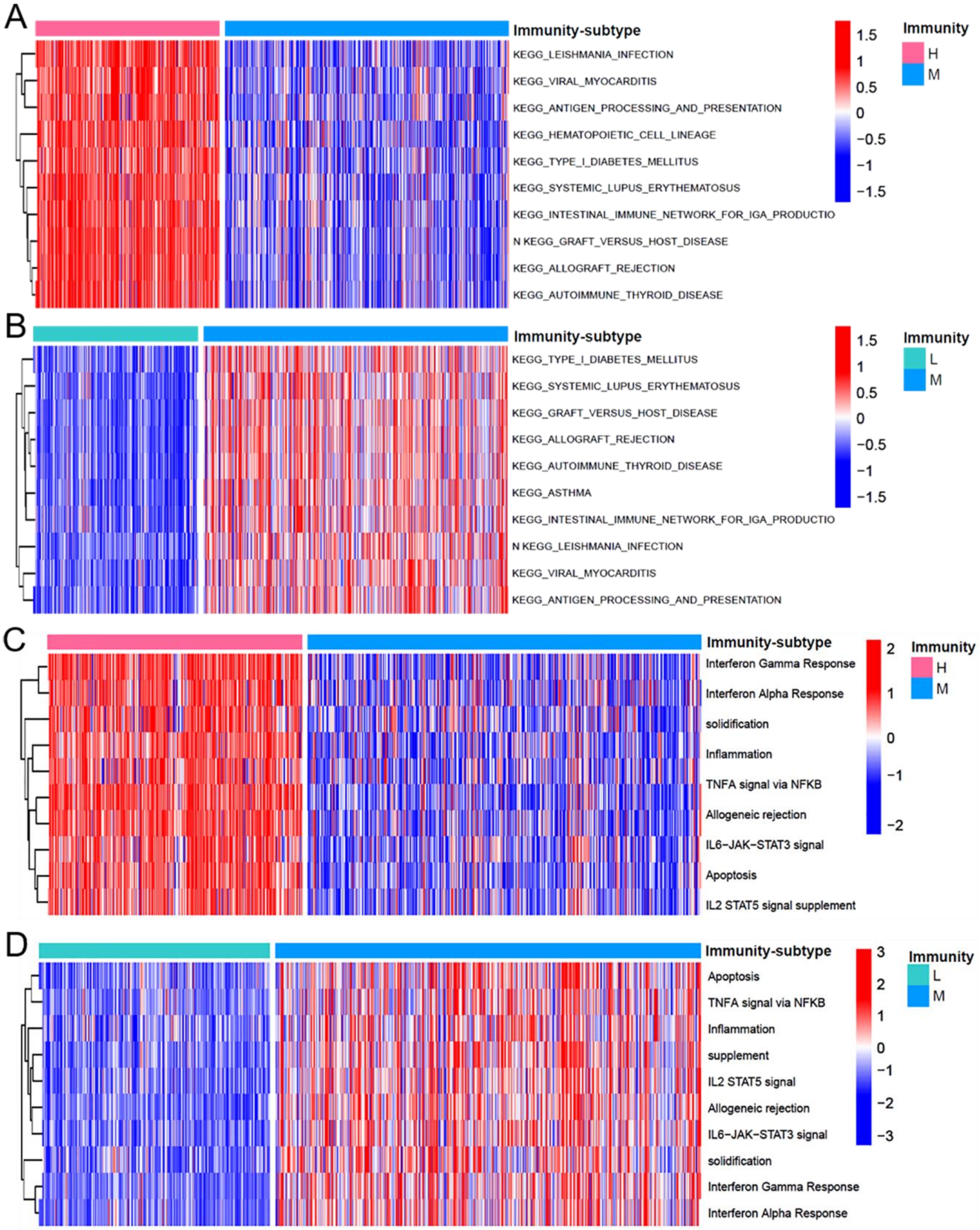

Figure S4. Enrichment analysis of glioma immune subtype related pathways. (A) Heatmap shows the GSVA score of top 10 KEGG pathways curated from MSigDB between immunity-H and immunity-M subtypes. (B) Heatmap shows the GSVA score of top 10 KEGG pathways curated from MSigDB between immunity-L and immunity-M subtypes. (C) Heatmap shows the GSVA score of top 10 hallmarks pathways curated from MSigDB between immunity-H and immunity-M subtypes. (D) Heatmap shows the GSVA score of top 10 hallmarks pathways curated from MSigDB between immunity-L and immunity-M subtypes.

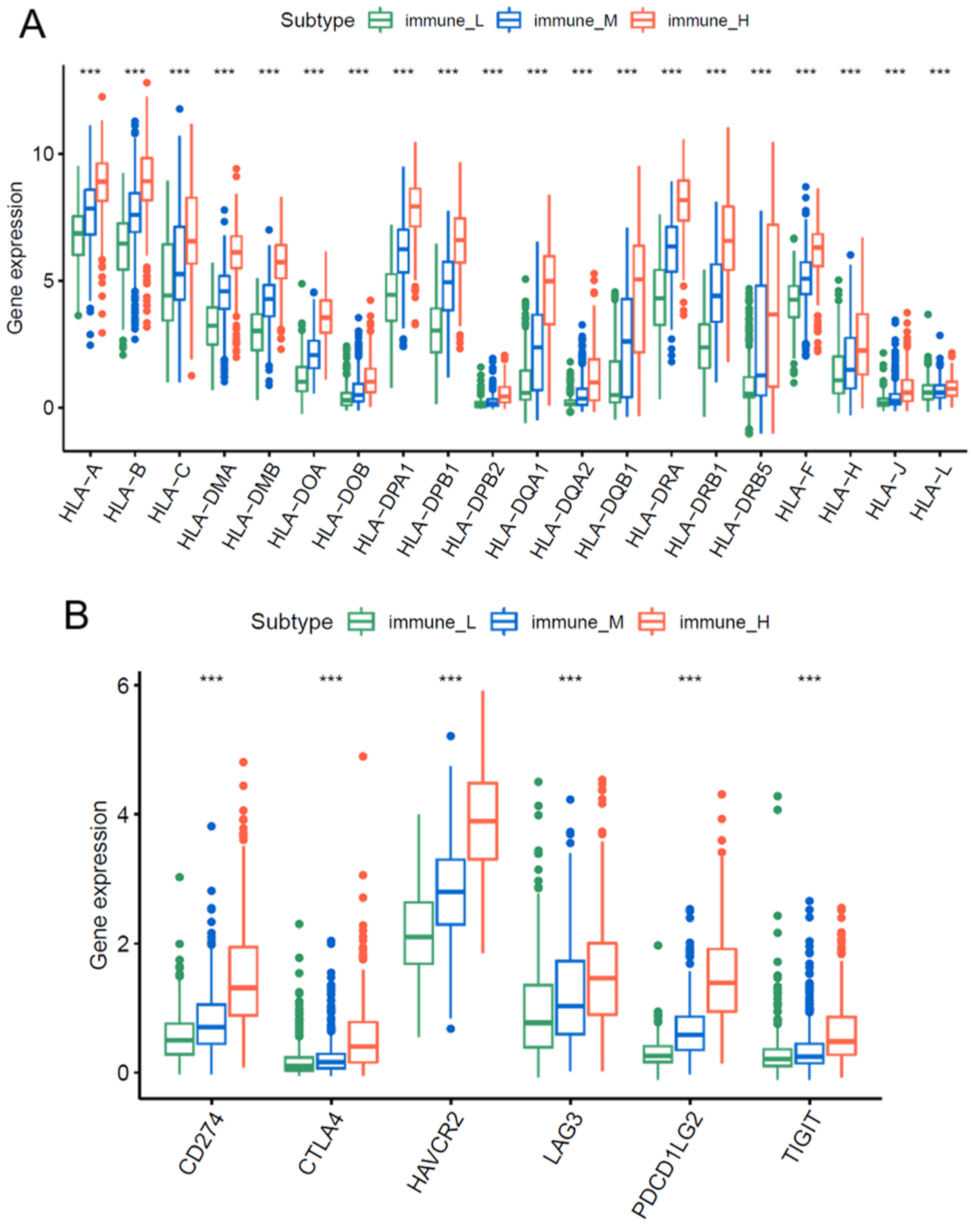

Figure S5. Comparison of HLA and ICB related genes expression among three immunity subtypes. (A) The expression of each HLA genes in three immunity-subtypes. (B) The expression of 6 ICB genes in three immunity-subtypes.

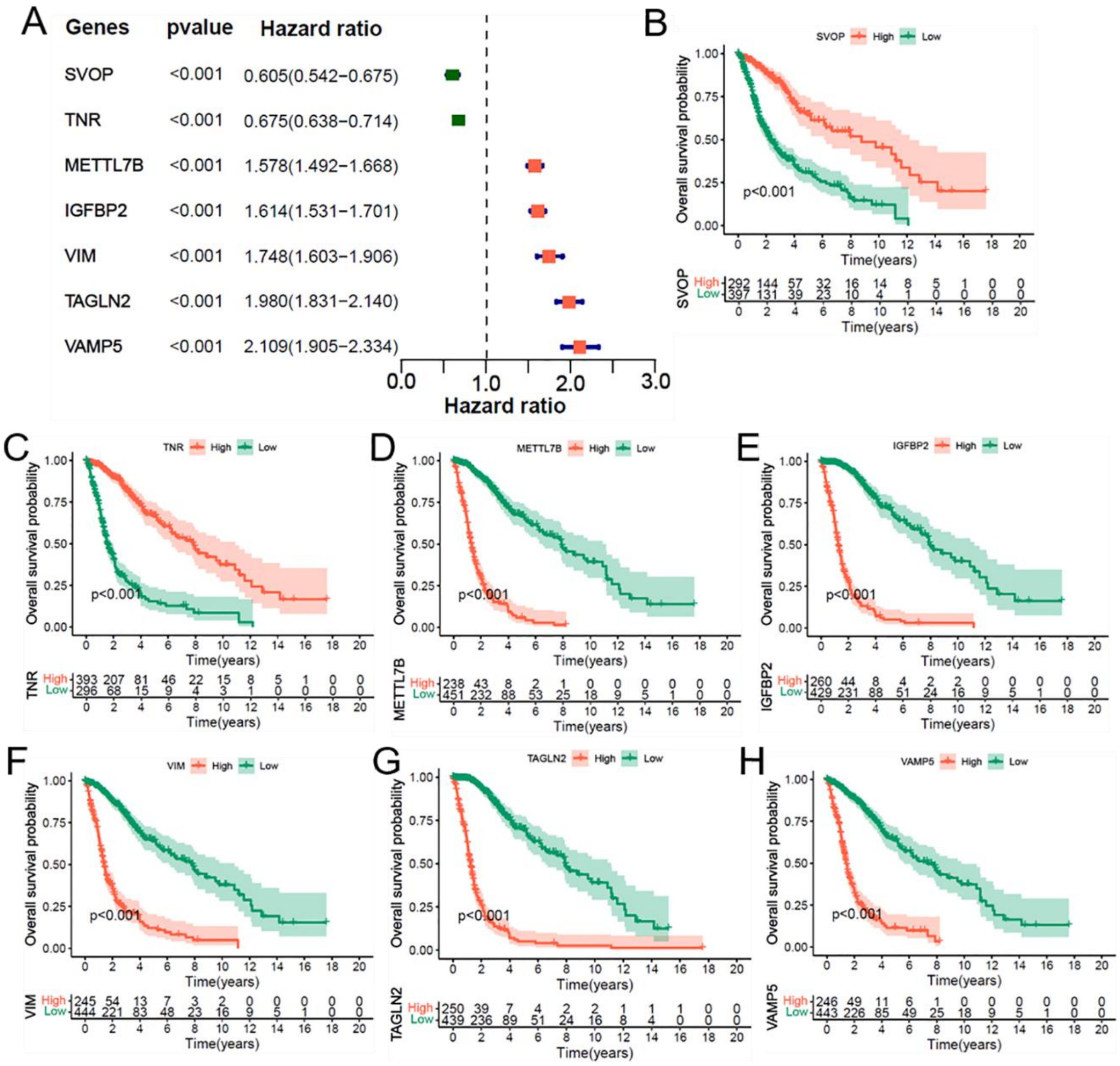

Figure S6. Univariate Cox Forest plot and Kaplan–Meier survival analysis of 7 hub genes in TCGA cohort. (A) Univariate Cox Forest plot of 7 immunity-subtype-related genes. (B) Kaplan–Meier curve survival analysis of 7 hub genes, (B) SVOP, (C) TNR, (D) VAMP5, (E) IGFBP2, (F) METTL7B, (G) VIM, (H) TAGLN2.

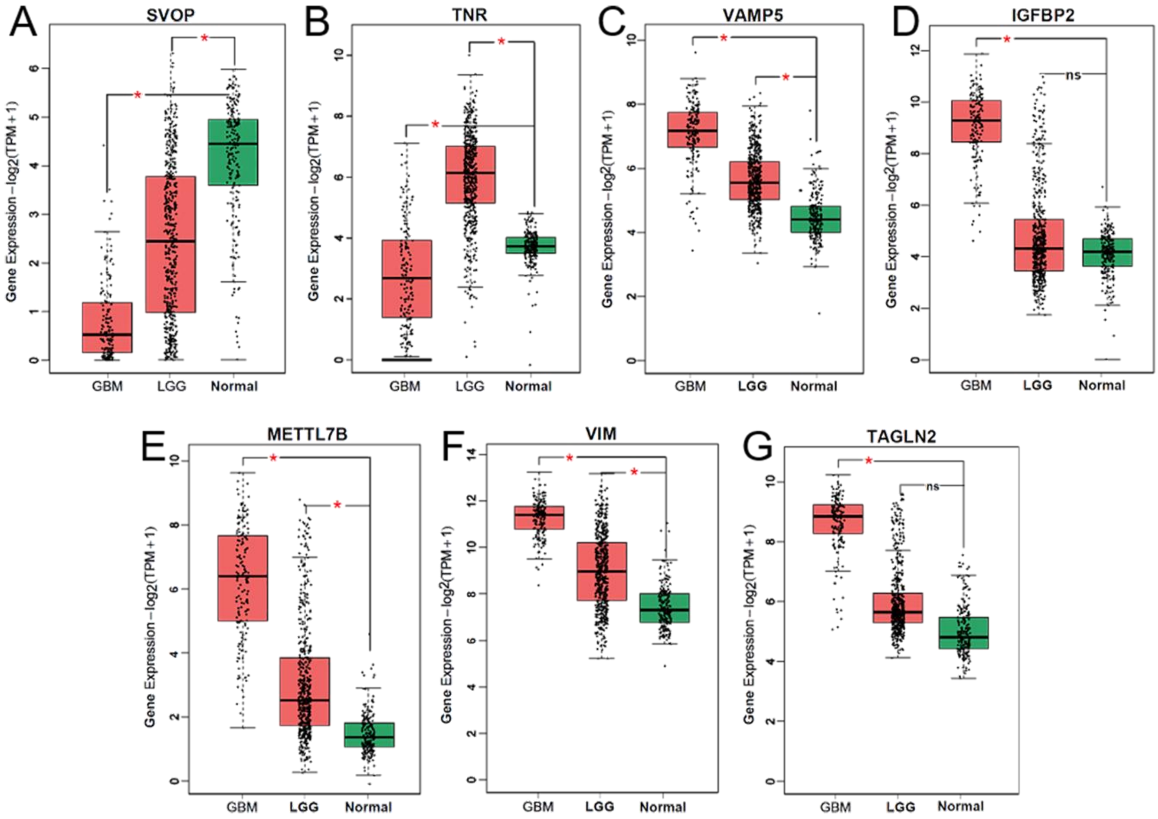

Figure S7. Comparison of 7 hub genes among GBM, LGG and normal tissue. (A-G) Comparison of 7 hub genes among GBM, LGG and normal tissue. (A) SVOP, (B) TNR, (C) VAMP5, (D) IGFBP2, (E) METTL7N, (F) VIM, (G) TAGLN2.

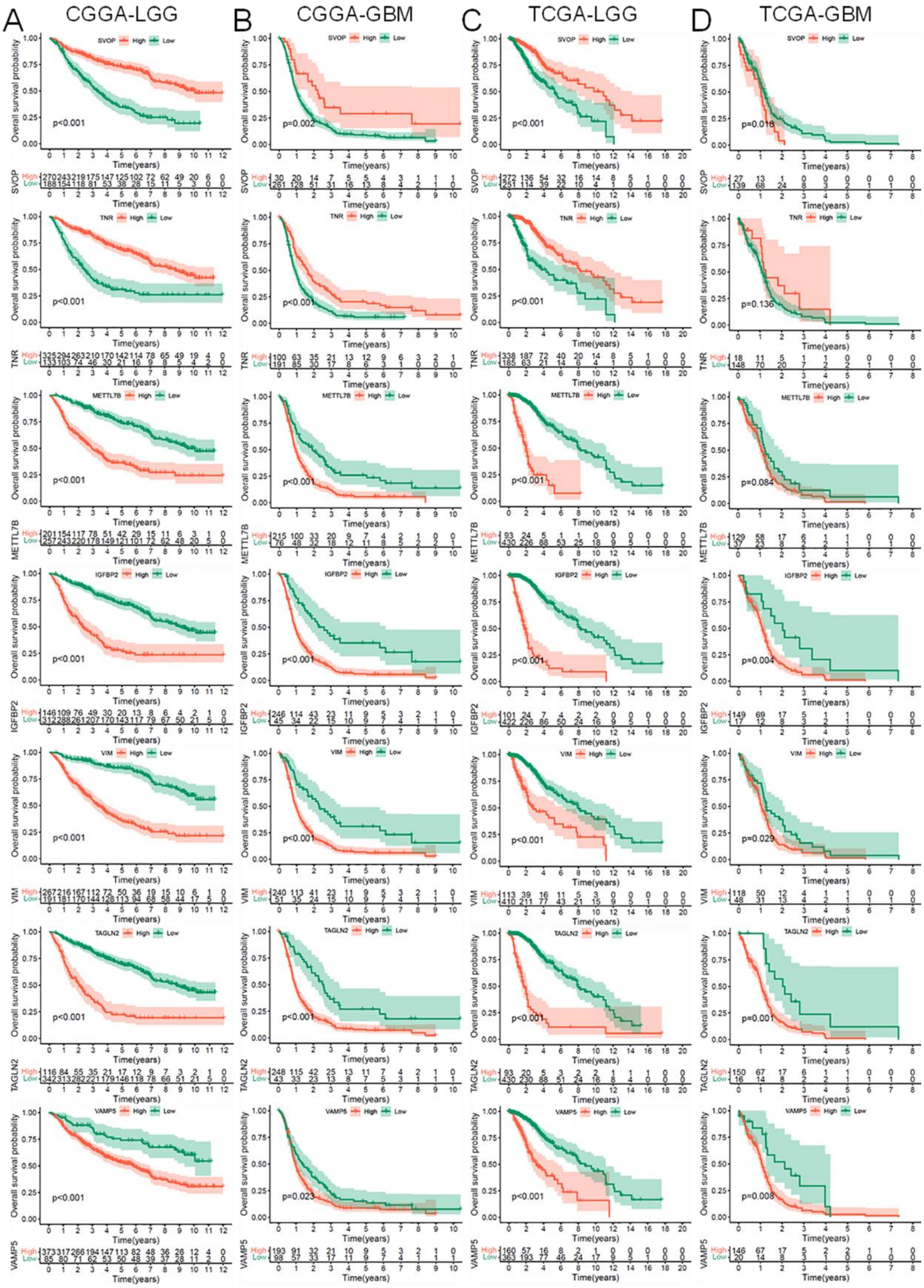

Figure S8. Prognostic survival analysis of seven core genes in PATIENTS with LGG and HGG glioma in CGGA and TCGA databases. Prognostic survival analysis of seven core genes in CGGA database(A-B) and TCGA database (C-D).
